# Free Volume and Gas Permeation in Anthracene Maleimide-Based Polymers of Intrinsic Microporosity

**DOI:** 10.3390/membranes5020214

**Published:** 2015-05-28

**Authors:** Muntazim Munir Khan, Volkan Filiz, Thomas Emmler, Volker Abetz, Toenjes Koschine, Klaus Rätzke, Franz Faupel, Werner Egger, Luca Ravelli

**Affiliations:** 1 Institute of Polymer Research, Helmholtz-Zentrum Geesthacht, Max-Planck-Str. 1, 21502 Geesthacht, Germany; E-Mails: muntazim.khan@hzg.de (M.M.K.); thomas.emmler@hzg.de (T.E.); volker.abetz@hzg.de (V.A.); 2 Institute of Physical Chemistry, University of Hamburg, Grindelallee 117, 20146 Hamburg, Germany; 3 Institute of Materials Science, University of Kiel, Technical Faculty, Chair for Multicomponent Materials, Kaiserstr. 2, 24143 Kiel, Germany; E-Mails: tk@tf.uni-kiel.de (T.K.); kr@tf.uni-kiel.de (K.R.); ff@tf.uni-kiel.de (F.F.); 4 Institut für Angewandte Physik und Messtechnik, Universität der Bundeswehr München, 85577 Neubiberg, Germany; E-Mails: werner.egger@unibw.de (W.E.); luca.ravelli@unibw.de (L.R.)

**Keywords:** polymer of intrinsic microporosity, free volume, antharcene maleimide, membranes, PALS

## Abstract

High free-volume copolymers were prepared via polycondensation with 2,3,5,6,-tetrafluoroterephthalonitrile (TFTPN) in which a portion of the 3,3,3',3'-tetramethyl-1,1'-spirobisindane (TTSBI) of PIM-1 was replaced with dibutyl anthracene maleimide (4bIII). An investigation of free volume using positron annihilation lifetime spectroscopy (PALS), and gas permeation measurements was carried out for the thin film composite copolymer membranes and compared to PIM-1. The average free volume hole size and the gas permeance of the copolymer membranes increased with decreasing TTSBI content in the copolymer.

## 1. Introduction

Membrane-based gas separation has attracted great interest in the industrial context due to its advantages over conventional separation methods. Polymeric materials are promising contender for fabricating membranes owing to the ease with which they are processed and their cost efficiency [1]. At present, only a few polymeric materials are used for industrial membrane-based separations, although a diverse number of materials have been reported for different gas separation pairs (e.g., O_2_/N_2_, CO_2_/N_2_, CO_2_/CH_4_) [2,3,4,5,6,7,8,9,10]. The achievement of high-efficiency gas separation requires high performance membranes which possess both good permeability and selectivity. However, there exists a trade-off between gas permeability and selectivity; this means highly permeable polymer membranes always display poor selectivity and *vice versa*. This trade-off has been described by the Robeson upper-bound relationship, which has been considered an empirical criterion for judging the broad spectrum of membranes [11].

In recent years, there have been several microporous organic polymers, such as solvent-swollen polymers [12,13], polymer networks with rigid units [14,15,16], and polymers with extremely bulky structural units [17,18] of considerable interest in terms of adsorbents, separation materials, and catalysis. This interest results from the combined advantages of polymer processability and high internal surface area, which is comparable with conventional inorganic microporous materials [19]. However, only a very limited number of microporous and soluble polymers are known so far. In 2004, Budd, McKeown, and coworkers reported a novel class of polymeric microporous material—polymers of intrinsic microporosity (PIMs) [20,21,22]. The intrinsic microporosity is caused by a rigid and contorted molecular structure which cannot fill space efficiently, leaving molecular-sized interconnected voids. This rigidity and contortion arises from the incorporation of non-linear “sites of contortion” such as spirocyclic or triptycene units [23,24]. Generally PIMs, especially PIM-1, can be easily prepared by step-growth polymerization between commercially available 5,5',6,6'-tetrahydroxy-3,3,3',3'-tetramethylspirobisindane (TTSBI) and 2,3,5,6,-tetrafluoroterephthalonitrile (TFTPN) [21].

There has been considerable research into the prototype PIM-1 and its analogues [25,26,27,28,29,30,31,32,33,34,35]. Significant improvements were obtained by recently developed Tröger’s base, sprobifluorene and triptycene-based ladder polymers, which exhibited prominent gas separation performance for various gas pairs such as O_2_/N_2_, H_2_/N_2_ and CO_2_/N_2_ with properties well above the 2008 Robeson upper bound curves [26,35,36].

**Figure 1:**
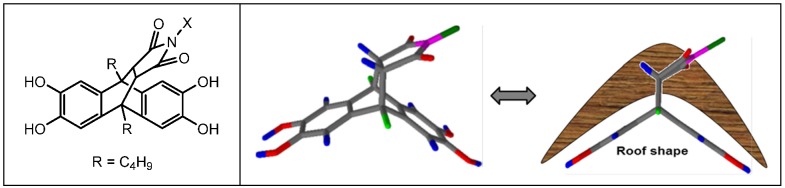
Anthracene maleimide comonomer structure.

As already pointed out in previous studies, the monomers such as ethanoanthracene or dialkyl anthracene maleimide, instead of the tetrahydroxy spirobisindane, may be also used to yield PIMs [37,38]. The homopolymers and copolymers obtained from dibutyl anthracene maleimide were soluble in most of the organic solvents and are characterized as “roof-shaped” resulting from the maleimide bridge over the anthracene ring (Figure 1). Anthracene maleimide introduces a “bend” into the polymer structure, and it is expected to be less flexible than a spiro-center, which involves a single tetrahedral carbon atom. This could modify the packing of the polymer chains and so affect the free volume in the desired way.

**Figure 2:**
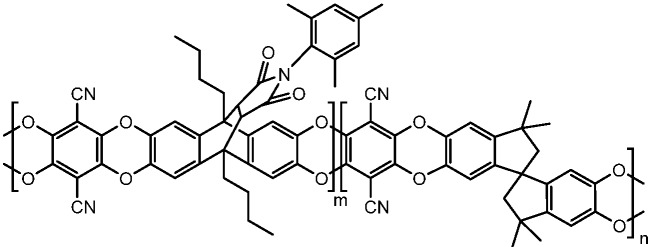
Homopolymer (*m*:*n* = 1:0) and copolymer (*m*:*n* = 3:1, 1:1, 1:2.3) of comonomer 4bIII.

From our previous studies [37], the copolymer of anthracene maleimide obtained from the comonomer 4b-III (Figure 2) shows a gas separation performance comparable to PIM-1. Motivated by this result, to fully understand the properties of the copolymers, we investigated the free volume behavior of these copolymers using positron annihilation lifetime spectroscopy (PALS) to clearly correlate the increase in permeability with the change in free volume and average hole size.

PALS probes the electron density distribution in materials via the measurement of the lifetime of a positron, between injection of the positron into the material and its decay with an electron of the sample. In insulating materials, ortho-Positronium (o-Ps) is formed, and its lifetime can be correlated to the average hole size via a well-established model [39]. The aim of this study is to compare the copolymers of comonomer 4bIII with respect to gas separation performance and free volume and to correlate the results.

## 2. Experimental Section

### 2.1. Materials

5,5',6,6'-tetrahydroxy-3,3,3',3'-tetramethyl-1,1'-spirobisindane (TTSBI, 97%) was obtained from ABCR (Karlsruhe, Germany), and 2,3,5,6,-tetrafluoro-terephthalonitrile (TFTPN, 99%) was kindly donated by Lanxess (Cologne, Germany). TFTPN was sublimated twice in vacuum before use. Potassium carbonate (K_2_CO_3_, >99.5%) was dried overnight under vacuum at 120 °C and milled in a ball mill for 15 min. Diethylbenzene (DEB) (isomeric mixture) was purchased from Sigma-Aldrich (Steinheim, Germany), and dimethyl acetamide (DMAc, ≥99%), tetrahydrofurane (THF, ≥99,9%), methanol (≥99.9%), chloroform (CHCl_3_, 99.99%), and dioxane (≥99%) from Merck (Darmstadt, Germany) were used as received.

### 2.2. Synthesis of Polymers

#### 2.2.1. Synthesis of Polymer of Intrinsic Microporosity (PIM-1)

TTSBI and TFTPN were dissolved in DMAc to form a homogeneous solution in a three-necked flask equipped with magnetic stirrer and argon inlet. Addition of K_2_CO_3_ (in excess of 1:1.2 based on OH-components) caused a colour change in the solution from yellow to orange. The reaction mixture flask was immersed in an oil bath preheated at 150 °C and within minutes the reaction mixture became increasingly viscous; then, DEB was added dropwise to maintain effective stirring, and stirring was continued for another 30 min. The hot reaction mixture was poured into methanol and the precipitated yellow solid was collected by filtration. The crude polymer was refluxed for 1 h in deionized water, filtrated and dried under vacuum at 120 °C for 48 h. The polymer was dissolved in chloroform and reprecipitated in methanol. In case the polymer product was insoluble in chloroform and dichlorobenzene, it was purified by washing with acetone [40,41,42,43].

#### 2.2.2. Synthesis of Anthracene Maleimide-Based Homopolymers and Copolymers

The synthesis and characterization of dialkyl anthracene maleimide comonomers [4a (I to V) and 4b (I to V)] was reported elsewhere [37]. For this study, comonomer 4b-III was used for further investigation. The series of homopolymers and copolymers was synthesized by polycondensation of comonomers with TFTPN and comonomers/TTSBI (different mol ratio) with TFTPN, respectively, using a procedure similar to that of PIM-1 (as described in Section 2.2.1). The homopolymers are referred to as PIM-4bIII-100 and the copolymers are identified as PIM-4bIII-75, 50 and 30, where PIM stands for polymer of intrinsic microporosity and suffixes -100, -75, -50, -30 refer to the mol% of comonomer in the copolymer. The apparent surface area of the polymer was calculated from N_2_ adsorption data via multipoint BET analysis. Molar mass distribution of the polymers was determined via gas permeation chromatography (GPC) with chloroform as solvent using polystyrene calibrated standards [37].

### 2.3. Membrane Preparation

Thin film composite (TFC) membranes for gas permeation and positron annihilation spectroscopy (PALS) studies were prepared on a microporous polyacrylonitrile (PAN) support (made in-house, average pore size of 25 nm with 15% surface porosity) and polymer solution (1% w/w) in chloroform using a laboratory scale casting machine. The details of membrane fabrication conditions were published elsewhere [44].

### 2.4. Gas Permeation Measurement

The permeation test involved the use of a gas permeation cell in which the membrane was placed on a sintered metal plate and pressurized at the feed side (Feed pressure: 1 bar; Temperature: 25 °C). Gas permeation rates were determined by a constant pressure variable volume system using a BIOS Definer™ 220 flow meter. The details of the gas permeation test cell were published elsewhere [44]. The pressure-normalized gas permeation flux or permeance (*P*) for a gas *i* can be calculated as follows:

$$P_{i}=\frac{J_{i}l}{A\Delta p}$$

where *J_i_* is the volumetric flow rate of gas *i* (normal cubic meter per hour), Δ*p* is the pressure difference across the membrane (bar), *A* is the membrane-effective surface area (square meters) and *l* is the membrane separating layer thickness (centimeters). The ideal separation factor α*_i/j_* was calculated by using the following equation:

$$\alpha_{i/j}=\frac{P_{i}}{P_{j}}$$

### 2.5. Positron Annihilation Lifetime Spectroscopy (PALS)

Due to the relative small selective layer thickness (~780 nm) of the polymer a conventional PALS setup cannot be used. Therefore, all measurements were made at the pulsed low energy positron system (PLEPS) [45] of the neutron induced positron source (NEPOMUC) at the Heinz Maier-Leibnitz Zentrum (MLZ) in Garching, Germany [46]. All samples were measured at 2 keV and 4 keV positron implantation energy to ensure that all positrons annihilate in the sample layer and not in the substrate. The spectra contained at least 5 × 10^6^ counts each. All measurements were made at room temperature (~30 °C). Counting rates of 3500 cts/s were achieved at a time resolution of better than 300 ps and a peak to background ratio of at least 10,000 to 1.

For the evaluation of the PALS data, the program POSWIN was used [45]. In order to obtain a reliable result, several approaches for fitting were adopted. First, for each implantation energy, free fits for most parameters were used and the deviation between fit and data was reduced by increasing the number of possible lifetimes. Finally, averages for background and p-Ps lifetime were fixed and the corresponding results for the two long o-Ps lifetimes were obtained with higher reliability [47,48,49,50,51]. In the following, only the longest o-Ps lifetime τ_4_, which changes within the polymers, is depicted.

### 2.6. Scanning Electron Microscopy

A LEO 1550 VP (Zeiss) equipped with a field emission cathode operated at 1–1.5 kV was used to study the morphology of pure PIM-1 and PIM-copolymers TFC membranes. For cross section analysis, the samples were fractured cryogenically in liquid nitrogen to get a distinct view of the membrane’s selective layer section. Before scanning, the membrane samples were coated with Pt with a sputter coater.

## 3. Results and Discussion

### 3.1. Synthesis of Polymers

**Figure 3:**
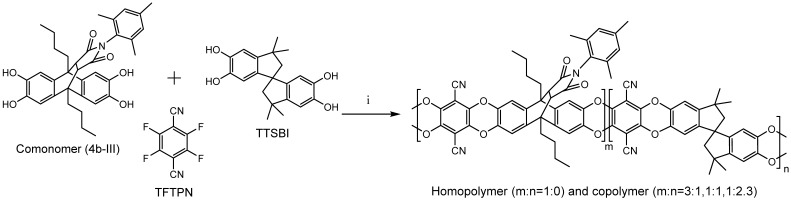
Synthesis of the homopolymer and copolymer of 4b-III (i = K_2_CO_3_, DMAc, DEB, 150 °C, 30 min).

The comonomer synthesis using a multistep synthetic route and the characterization were described elsewhere [37]. The homopolymer and copolymers were synthesized via aromatic nuleophilic polycondensation reaction by reacting tetrahydroxy monomers with equimolar amounts of TFTPN, catalyzed by an excess of K_2_CO_3_, using a modified procedure similar to the synthesis of PIM-1 (Figure 3) [37,51].

It is known from a previous study that the byproducts like cyclic oligomers and structural cross-linking can be effectively reduced by using high temperature and high monomer concentrations as polycondensation reaction conditions [52]. In the present work, a slightly modified polymerization procedure [53] was used based on a previously reported polymerization reaction [52]. DEB which is advantageous not only to provide solubility enhancement for the polymer but also to provide a high-boiling point solvent to the reaction was used in the polymerization reaction instead of toluene. To keep the reaction mixture less viscous, additional amounts of DEB were added. The presence of DEB required precipitation of the polymers in methanol. It should be noted that polymers obtained under this condition produce high-molecular-weight polymers with a broad range of polydispersity, which is typical for polycondensation reactions. Table 1 reports the molecular weights, polydispersities (as determined by gel permeation chromatography (GPC) against polystyrene standards) and surface area of homopolymer and copolymers.

**Table 1:** Physical properties of homopolymers, copolymers and PIM-1.

Polymers	Comonomer (mol ratio)	TTSBI (mol ratio)	TFTPN (mol ratio)	Mw (g/mol)	PDI	Surface Area (m^2^/g)
PIM-4bIII-100	1	0	1	80200	4.2	660
PIM-4bIII-75	3	1	4	93300	3.8	790
PIM-4bIII-50	1	1	2	147300	6.2	870
PIM-4bIII-30	1	2.3	3.3	118700	5.4	850
PIM-1	0	1	1	221000	4.8	830

Thus, under the same reaction conditions (150 °C, 30 min), molecular weight broadened by introducing a certain ratio of comonomer into the polymer chain, and high-molecular-weight copolymers could be obtained. A plausible explanation could be the different reactivity of the monomers and their different mole ratio in the reaction mixture. As shown in Table 1, compared to PIM-1, BET surface area of the homopolymer (PIM-4bIII-100) decreased from 830 m^2^/g for PIM-1 to 660 m^2^/g for the homopolymer. The reason could be a stronger cohesive interaction between the polymer chains due to the polar imido group, or occlusion of maleimide derivatives in the micropores. The BET surface area for copolymers with different monomer ratios (4b-III) is comparable to or higher than PIM-1 (Table 1). The introduction of the anthracene maleimide segment into PIMs could change the extending state of the polymer chains. Rigid molecular conformation of anthacene maleimide units probably frustrates the effective folding of polymer chains, which results in a high surface area for anthracene maleimide-containing PIM.

### 3.2. Cross Section Morphology

**Figure 4:**
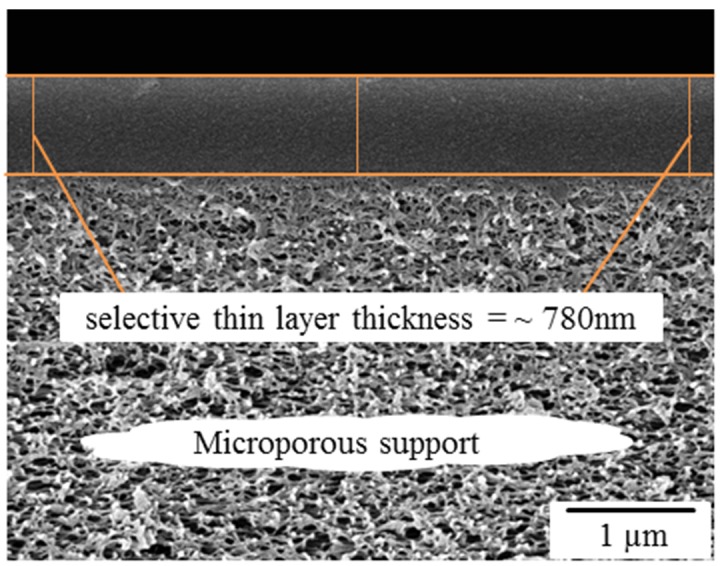
Cross section SEM image of PIM-1 polymer thin-film composite membrane.

Figure 4 shows the scanning electron microscope (SEM) image of a cross section of a PIM-1 thin-film composite membrane. The thickness of the thin selective polymer layer on the microporous PAN support was calculated from the cross section of the membranes. It was observed that the average thickness of all copolymers membranes was ~780 nm.

### 3.3. Gas Permeation Performance

Physisorption and pore size distribution measurements revealed the influence of the polymeric microstructure of the anthracene substituted maleimide (4bIII) moiety on the gas transport properties [37]. Pure gas permeance for N_2_, O_2_, and CO_2_ was measured at 25 °C by our home-built permeation test facility [44]. Table 2 shows the permeances for PIM-1, homopolymer and copolymers thin film composite membranes. All the data presented in this part were obtained from at least three stamps of thin film composites membranes.

**Table 2:** Gas permeance of PIM-1, homopolymer and copolymers.

Polymer Samples	Permeance (Nm^3^/m^2^ h bar)		
	N_2_	O_2_	CO_2_
PIM-4bIII-100	1.86 (±0.18)	4.60 (±0.21)	34.23 (±0.25)
PIM-4bIII-75	1.74 (±0.15)	4.48 (±0.14)	33.41 (±0.26)
PIM-4bIII-50	1.71 (±0.16)	4.45 (±0.11)	33.24 (±0.21)
PIM-4bIII-30	1.70 (±0.14)	4.32 (±0.10)	32.84 (±0.22)
PIM-1	1.69 (±0.20)	4.89 (±0.19)	34.66 (±0.23)

The copolymer obtained from comonomer 4bIII shows a similar permeance compared to a PIM-1 thin film composite membrane. The order of gas permeance was observed as CO_2_ > O_2_ > N_2_. Compared to PIM-1, which was tested under the same conditions, PIM-4bIII-copolymers have slightly lower O_2_/N_2_ and CO_2_/N_2_ permselectivity. (Table 3).

**Table 3:** Permselectivity of PIM-1, homopolymer and copolymers.

Polymer Samples	Permselectivity	
	O_2_/N_2_	CO_2_/N_2_
PIM-1	2.9	20.5
PIM-4bIII-100	2.5	18.4
PIM-4bIII-75	2.6	19.2
PIM-4bIII-50	2.6	19.4
PIM-4bIII-30	2.5	19.3

The gas separation performance of the copolymer increased slightly with increasing TTSBI content. The reason for this could be the hindered rotation of the ortho-substituted aniline group. Moreover, the high surface area of the copolymer, pore size distribution and the rigid three-dimensional architecture of 4bIII could be another reason for the increasing permeances.

From a material and structural viewpoint, a smaller interchain distance combined with greater chain rigidity contributes to an increased selectivity but lower permeances, whereas longer chain distances contribute to higher permeances but lower permselectivity.

### 3.4. Positron Annihilation Lifetime Spectroscopy (PALS)

Many properties of the polymers, such as glass transition temperature, permeability and diffusivity, are affected by the free volume, which to a first approach, is the unoccupied space between the molecules. There are several methods reported for the investigation of the free volume in polymers (e.g., pressure-volume-temperature (PVT), ^129^Xe NMR spectroscopy, and PALS). Whereas pressure–volume–temperature measurements give information exclusively about the total free volume and its temperature dependence, both ^129^Xe NMR spectroscopy and PALS [38,54] measurements provide information about the average microscopic size of free volume entities. A comparison of selected techniques concerning investigations of the free volume of polymers was reported elsewhere [55]. In this work, PALS was used to determine the free volume of polymers. Once injected from a radioactive source, positrons form hydrogen-like positronium states in most polymers. The pick-off annihilation of the o-Ps depends on the local electron density distribution around the o-Ps and thus the o-Ps lifetime can be used to determine the average free volume hole size radius. This is usually done by applying a standard quantum mechanical model originally developed by Tao and later expanded on by Eldrup and Jean, as discussed in many textbooks [39]. In this model, the o-Ps is assumed to be confined in a spherical potential well with infinite walls, which are “decorated” with an electron layer at its wall where it can decay by pick-off annihilation. The calculation of the overlap integral of the positronium probability density function and the electron layer results in a direct relation between the o-Ps lifetime τ_o-Ps_ and the average free volume hole radius *R*:

$$\frac{1}{\tau_{o-Ps}}=\lambda_{0}\left(1-\frac{R_{h}}{R_{h}+\delta R}+\frac{1}{2\pi }\sin\frac{2\pi R_{h}}{R_{h}+\delta R}\right)$$

This equation includes the reciprocal o-Ps decay rate τ_-o-Ps,_ the spin averaged decay rate in the electron layer at the edge of the potential well λ_0_, the thickness of the electron layer δR = 0.166 nm as determined by using materials with a well-known pore size [56], and the average free volume hole radius R_h_. Additional information can be obtained from the intensities, which are the relative probabilities of the three decay possibilities (para-positronium, free positron, ortho-positronium) discussed in the previous paragraph. The positronium intensities (o-Ps and p-Ps) depend on the formation probability of positronium in the respective polymeric materials, which are not known *a priori* and are often also related to the hole concentration [39]. Additionally, the measured o-Ps intensity is proportional to the amount of polymer “seen” by the positrons, which is particularly important for the thin layers investigated here.

In order to investigate the influence of polymer structure on the hole size of the free volume sites, different thin-film polymer composite membranes were prepared with variations in the comonomer concentration. For example the PIM-4bIII-30 to PIM-4bIII-100 has different mol% incorporation in the polymer backbone. Since the TTSBI group has a rigid contorted structure, it was expected that the packing density would be reduced with increasing TTSBI content in the polymer structure. However, the anthracene maleimide sturucture provides a bulky substituent on anthracene and a roof-shaped structure, resulting in a more compact polymer chain, and packing density could be increased by reducing the antharcene maleimide content in the polymer structure. Along with the increasing TTSBI mol% in the copolymer, the comparative gas permeances were observed in the copolymer membranes. The permeation experiments only yielded information on the overall free volume of the different structures. No information on the changes in free volume sites dependent on structural variation could be obtained. Therefore, PALS experiments were used to track the changes of the free volume-dependent hole size in the polymer structure.

**Figure 5:**
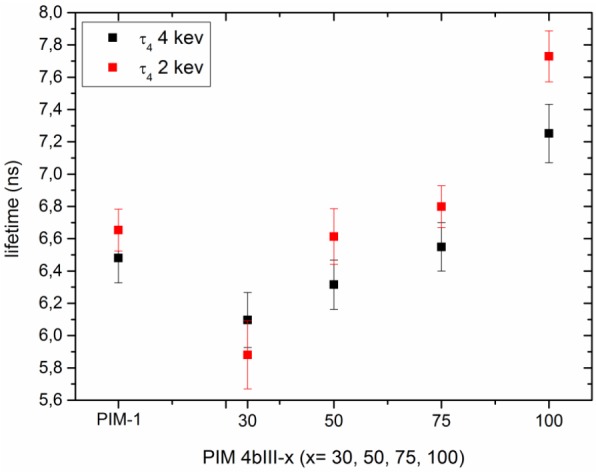
o-Ps lifetime as a function of polymer variants (4bIII).

Figure 5 shows the o-Ps lifetime τ4 obtained from the PALS experiments for different thin-film polymer samples. The o-Ps lifetimes 6 and 7.5 ns correspond to hole diameters of 1.04 and 1.15 nm, respectively. As TTSBI content in the copolymer decreases, the average free volume increases, which again shows the general reliability of the technique.

**Figure 6:**
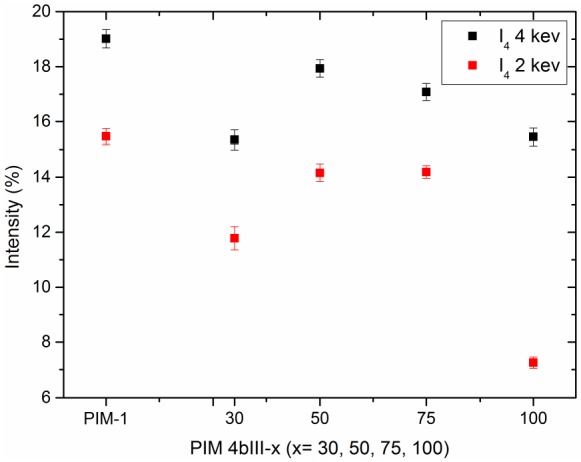
o-Ps intensities corresponding to o-Ps lifetimes in Figure 5 as a function of polymer variants (4bIII).

Figure 6 shows the intensities corresponding to the o-Ps lifetimes in Figure 5, which is proportional to the τ_o-Ps_ formation probability multiplied by the hole concentration. The o-Ps intensities do not show significant tendencies with the polymer variants. However, the data point for 100% and 2 keV is significantly lower, and the reason for this is not clear. Regardless, this does not affect the corresponding o-Ps lifetime.

Comparing the free volume and the gas permeance measurement, one could see that the incorporation of the comonomer into the copolymer slightly increases the permeance compared to pure PIM-1. The permeance increment coupled with lower permselectivity for the copolymer membrane can be explained by the polymer chain packing. This means that the packing is disturbed in the sense of a more disordered arrangement of the polymer chains. This is also seen in the PALS measurement that showed an increase in hole size as well as (slightly) higher free volume with respect to the rising comonomer content in the copolymer. The enhanced gas permeance of the copolymers correlates well with our PALS results, which show an increase in the free volume compared to PIM-1 membranes.

## 4. Conclusions

Various copolymers were synthesized in which a portion of the spiro unit (TTSBI) of PIM-1 was replaced by antharcene maleimide-based comonomers (4bIII). Free volume and gas permeance measurement were carried out with copolymers obtained from comonomer 4bIII and compared to PIM-1. PALS measurements show that the hole size of the free volume sites of the copolymers increased with decreasing TTSBI content in the copolymer. Gas permeance of the copolymers was slightly higher compared to PIM-1, coupled with no significant reduction of the permselectivities. These results suggest that these copolymers are potential candidates for gas separation membrane materials and have a good combination of physical properties, similar to PIM-1.

## References

[1] Pandey P., Chauhan R.S. (2001). Membranes for gas separation. Prog. Polym. Sci..

[2] Rahman M.M., Filiz V., Shishatskiy S., Abetz C., Neumann S., Bolmer S., Khan M.M., Abetz V. (2013). Pebax^®^ with peg functionalized poss as nanocomposite membranes for CO_2_ separation. J. Membr. Sci..

[3] Rahman M.M., Shishatskiy S., Abetz C., Georgopanos P., Neumann S., Khan M.M., Filiz V., Abetz V. (2014). Influence of temperature upon properties of tailor-made pebax^®^ mh 1657 nanocomposite membranes for post-combustion CO_2_ capture. J. Membr. Sci..

[4] Rahman M.M., Filiz V., Khan M.M., Gacal B.N., Abetz V. (2015). Functionalization of poss nanoparticles and fabrication of block copolymer nanocomposite membranes for CO_2_ separation. React. Funct. Polym..

[5] Rahman M.M., Filiz V., Shishatskiy S., Abetz C., Georgopanos P., Khan M.M., Neumann S., Abetz V. (2015). Influence of poly(ethylene glycol) segment length on CO_2_ permeation and stability of polyactive membranes and their nanocomposites with PEG POSS. ACS Appl. Mater. Interfaces.

[6] George S.C., Thomas S. (2001). Transport phenomena through polymeric systems. Prog. Polym. Sci..

[7] Maier G. (1998). Gas separation with polymer membranes. Angew. Chem. Int. Edit..

[8] Swaidan R., Ghanem B., Litwiller E., Pinnau I. (2015). Effects of hydroxyl-functionalization and sub-*T_g_* thermal annealing on high pressure pure- and mixed-gas CO_2_/CH_4_ separation by polyimide membranes based on 6FDA and triptycene-containing dianhydrides. J. Membr. Sci..

[9] Yampolskii Y. (2012). Polymeric gas separation membranes. Macromolecules.

[10] Baker R.W., Low B.T. (2014). Gas separation membrane materials: A perspective. Macromolecules.

[11] Robeson L.M. (2008). The upper bound revisited. J. Membr. Sci..

[12] Hradil J., Švec F. (1990). Reactive polymers. 61. Synthesis of strongly basic anion exchange methacrylate resins. React. Polym..

[13] Tsyurupa M.P., Davankov V.A. (2002). Hypercrosslinked polymers: Basic principle of preparing the new class of polymeric materials. React. Funct. Polym..

[14] McKeown N.B., Gahnem B., Msayib K.J., Budd P.M., Tattershall C.E., Mahmood K., Tan S., Book D., Langmi H.W., Walton A. (2006). Towards polymer-based hydrogen storage materials: Engineering ultramicroporous cavities within polymers of intrinsic microporosity. Angew. Chem. Int. Ed..

[15] Webster O.W., Gentry F.P., Farlee R.D., Smart B.E. (1992). Hypercrosslinked rigid-rod polymers. Makromol. Chem. Macromol. Symp..

[16] Wood C.D., Tan B., Trewin A., Niu H., Bradshaw D., Rosseinsky M.J., Khimyak Y.Z., Campbell N.L., Kirk R., Stöckel E. (2007). Hydrogen storage in microporous hypercrosslinked organic polymer networks. Chem. Mater..

[17] Dai Y., Guiver M.D., Robertson G.P., Kang Y.S. (2005). Effect of hexafluoro-2-propanol substituents in polymers on gas permeability and fractional free volume. Macromolecules.

[18] Dai Y., Guiver M.D., Robertson G.P., Kang Y.S., Lee K.J., Jho J.Y. (2004). Preparation and characterization of polysulfones containing both hexafluoroisopropylidene and trimethylsilyl groups as gas separation membrane materials. Macromolecules.

[19] Du N., Robertson G.P., Pinnau I., Thomas S., Guiver M.D. (2009). Copolymers of intrinsic microporosity based on 2,2',3,3'-tetrahydroxy-1,1'-dinaphthyl. Macromol. Rapid Commun..

[20] Ghanem B.S., Msayib K.J., McKeown N.B., Harris K.D.M., Pan Z., Budd P.M., Butler A., Selbie J., Book D., Walton A. (2007). A triptycene-based polymer of intrinsic microposity that displays enhanced surface area and hydrogen adsorption. Chem. Commun..

[21] Budd P.M., Elabas E.S., Ghanem B.S., Makhseed S., McKeown N.B., Msayib K.J., Tattershall C.E., Wang D. (2004). Solution-processed, organophilic membrane derived from a polymer of intrinsic microporosity. Adv. Mater..

[22] McKeown N.B., Budd P.M., Msayib K.J., Ghanem B.S., Kingston H.J., Tattershall C.E., Makhseed S., Reynolds K.J., Fritsch D. (2005). Polymers of intrinsic microporosity (PIMs): Bridging the void between microporous and polymeric materials. Chem. A Eur. J..

[23] Carta M., Msayib K.J., Budd P.M., McKeown N.B. (2008). Novel spirobisindanes for use as precursors to polymers of intrinsic microporosity. Org. Lett..

[24] Budd P.M., Ghanem B.S., Makhseed S., McKeown N.B., Msayib K.J., Tattershall C.E. (2004). Polymers of intrinsic microporosity (PIMs): Robust, solution-processable, organic nanoporous materials. Chem. Commun..

[25] Du N., Robertson G.P., Dal-Cin M.M., Scoles L., Guiver M.D. (2012). Polymers of intrinsic microporosity (PIMs) substituted with methyl tetrazole. Polymer.

[26] Bezzu C.G., Carta M., Tonkins A., Jansen J.C., Bernardo P., Bazzarelli F., McKeown N.B. (2012). A spirobifluorene-based polymer of intrinsic microporosity with improved performance for gas separation. Adv. Mater..

[27] Du N., Dal-Cin M.M., Robertson G.P., Guiver M.D. (2012). Decarboxylation-induced cross-linking of polymers of intrinsic microporosity (PIMs) for membrane gas separation. Macromolecules.

[28] Ma X., Salinas O., Litwiller E., Pinnau I. (2013). Novel spirobifluorene- and dibromospirobifluorene-based polyimides of intrinsic microporosity for gas separation applications. Macromolecules.

[29] Rogan Y., Starannikova L., Ryzhikh V., Yampolskii Y., Bernardo P., Bazzarelli F., Jansen J.C., McKeown N.B. (2013). Synthesis and gas permeation properties of novel spirobisindane-based polyimides of intrinsic microporosity. Polym. Chem..

[30] Carta M., Malpass-Evans R., Croad M., Rogan Y., Jansen J.C., Bernardo P., Bazzarelli F., McKeown N.B. (2013). An efficient polymer molecular sieve for membrane gas separations. Science.

[31] Del Regno A., Gonciaruk A., Leay L., Carta M., Croad M., Malpass-Evans R., McKeown N.B., Siperstein F.R. (2013). Polymers of intrinsic microporosity containing tröger base for CO_2_ capture. Ind. Eng. Chem. Res..

[32] Shamsipur H., Dawood B.A., Budd P.M., Bernardo P., Clarizia G., Jansen J.C. (2014). Thermally rearrangeable PIM-polyimides for gas separation membranes. Macromolecules.

[33] Taylor R.G.D., Carta M., Bezzu C.G., Walker J., Msayib K.J., Kariuki B.M., McKeown N.B. (2014). Triptycene-based organic molecules of intrinsic microporosity. Org. Lett..

[34] Zhang J., Jin J., Cooney R., Zhang S. (2015). Synthesis of polymers of intrinsic microporosity using an ab-type monomer. Polymer.

[35] Carta M., Malpass-Evans R., Croad M., Rogan Y., Lee M., Rose I., McKeown N.B. (2014). The synthesis of microporous polymers using troger’s base formation. Polym. Chem..

[36] Ghanem B.S., Swaidan R., Litwiller E., Pinnau I. (2014). Ultra-microporous triptycene-based polyimide membranes for high-performance gas separation. Adv. Mater..

[37] Khan M.M., Bengtson G., Neumann S., Rahman M.M., Abetz V., Filiz V. (2014). Synthesis, characterization and gas permeation properties of anthracene maleimide-based polymers of intrinsic microporosity. RSC Adv..

[38] Emmler T., Heinrich K., Fritsch D., Budd P.M., Chaukura N., Ehlers D., Rätzke K., Faupel F. (2010). Free volume investigation of polymers of intrinsic microporosity (PIMs): PIM-1 and PIM1 copolymers incorporating ethanoanthracene units. Macromolecules.

[39] Jean Y.C., Mallon P.E., Schrader D.M. (2003). Principle and Application of Positron and Positronium Chemistry.

[40] Koschine T., Rätzke K., Faupel F., Khan M.M., Emmler T., Filiz V., Abetz V., Ravelli L., Egger W. (2015). Correlation of gas permeation and free volume in new and used high free volume thin film composite membranes. J. Polym. Sci. Part B Polym. Phys..

[41] Khan M.M., Filiz V., Bengtson G., Shishatskiy S., Rahman M.M., Lillepaerg J., Abetz V. (2013). Enhanced gas permeability by fabricating mixed matrix membranes of functionalized multiwalled carbon nanotubes and polymers of intrinsic microporosity (PIM). J. Membrane Sci..

[42] Khan M.M., Bengtson G., Shishatskiy S., Gacal B.N., Mushfequr Rahman M., Neumann S., Filiz V., Abetz V. (2013). Cross-linking of polymer of intrinsic microporosity (PIM-1) via nitrene reaction and its effect on gas transport property. Eur. Polym. J..

[43] Khan M.M., Filiz V., Bengtson G., Rahman M.M., Shishatskiy S., Abetz V. (2012). Functionalized carbon nanotube mixed matrix membranes of polymers of intrinsic microporosity (PIMs) for gas separation. Procedia Eng..

[44] Khan M., Filiz V., Bengtson G., Shishatskiy S., Rahman M., Abetz V. (2012). Functionalized carbon nanotubes mixed matrix membranes of polymers of intrinsic microporosity for gas separation. Nanoscale Res. Lett..

[45] Egger W. (2010). Positron Sources and Positron Beams, Physics with Many Positrons.

[46] Ohrt C., Koschine T., Rätzke K., Faupel F., Willner L., Schneider G.J. (2014). Free volume in pep-silica nanocomposites with varying molecular weight. Polymer.

[47] Harms S., Rätzke K., Faupel F., Chaukura N., Budd P.M., Egger W., Ravelli L. (2012). Aging and free volume in a polymer of intrinsic microporosity (PIM-1). J. Adhes..

[48] Konietzny R., Koschine T., Rätzke K., Staudt C. (2014). Poss-hybrid membranes for the removal of sulfur aromatics by pervaporation. Sep. Purif. Technol..

[49] Harms S., Rätzke K., Pakula C., Zaporojtchenko V., Strunskus T., Egger W., Sperr P., Faupel F. (2011). Free volume changes on optical switching in azobenzene-polymethylmethacrylate blends studied by a pulsed low-energy positron beam. J. Polymer Sci. Part B Polym. Phys..

[50] Jeazet H., Koschine T., Staudt C., Raetzke K., Janiak C. (2013). Correlation of gas permeability in a metal-organic framework MIL-101(Cr)–polysulfone mixed-matrix membrane with free volume measurements by positron annihilation lifetime spectroscopy (PALS). Membranes.

[51] Fritsch D., Bengtson G., Carta M., McKeown N.B. (2011). Synthesis and gas permeation properties of spirobischromane-based polymers of intrinsic microporosity. Macromol. Chem. Phys..

[52] Du N., Song J., Robertson G.P., Pinnau I., Guiver M.D. (2008). Linear high molecular weight ladder polymer via fast polycondensation of 5,5',6,6'-tetrahydroxy-3,3,3',3'-tetramethylspirobisindane with 1,4-dicyanotetrafluorobenzene. Macromol. Rapid Commun..

[53] Fritsch D., Bengtson G., Carta M., McKeown N.B. (2011). Synthesis and gas permeation properties of spirobischromane-based polymers of intrinsic microporosity. Macromol. Chem. Phys..

[54] Suzuki T., Yamada Y. (2006). Characterization of 6FDA-based hyperbranched and linear polyimide–silica hybrid membranes by gas permeation and 129Xe NMR measurements. J. Polym. Sci. Part B Polym. Phys..

[55] Jansen J.C., Macchione M., Tocci E., de Lorenzo L., Yampolskii Y.P., Sanfirova O., Shantarovich V.P., Heuchel M., Hofmann D., Drioli E. (2009). Comparative study of different probing techniques for the analysis of the free volume distribution in amorphous glassy perfluoropolymers. Macromolecules.

[56] Schrader D.R., Jean Y.C. (1988). Positron and positronium in liquids. Positron and Positronium Chemistry.

